# Discussing psychological and psychotherapeutic support for patients with Marfan syndrome (MFS) and their family: an example of a structured program in Italy

**DOI:** 10.3389/fpsyg.2023.1176692

**Published:** 2023-06-15

**Authors:** Mariangela Panetta, Alessandra Bianchetti, Nathasha Samali Udugampolage, Jacopo Taurino, Rosario Caruso, Alessandro Pini, Edward Callus

**Affiliations:** ^1^Cardiovascular-Genetic Centre, IRCCS Policlinico San Donato, San Donato Milanese, Italy; ^2^Health Professions Research and Development Unit, IRCCS Policlinico San Donato, Milan, Italy; ^3^Department of Biomedical Sciences for Health, University of Milan, Milan, Italy; ^4^Clinical Psychology Service, IRCCS Policlinico San Donato, Milan, Italy

**Keywords:** cardiovascular disease, rare diseases, Marfan syndrome, psychological support, counseling

## 1. Introduction

Marfan syndrome (MFS) is a rare genetic disorder of the connective tissue caused by mutations in the Fibrillin-1 (FBN1) protein, which is encoded by the FBN1 gene (Loeys et al., 2010). The incidence of MFS is reported to be around 1 in 5,000 individuals, with no gender difference (Robinson et al., 2006; Pyeritz, 2019). MFS primarily affects the cardiovascular, ocular, and osteoarticular systems, with minor conditions affecting the lungs, dura, and skin (Loeys et al., 2010). Due to considerable phenotypic variability and overlap with other connective tissue diseases, a correct diagnosis of MFS is essential to prevent complications that can reduce life expectancy and cause sudden cardiac death (Loeys et al., 2010). For this reason, a correct diagnosis of MFS is extremely important for the patient since failure in treatment can result in a reduction in life expectancy and sudden cardiac death (Ghanta et al., 2016).

Aortic complications, such as dilation, dissection, and rupture of the ascending aorta, represent the major mortality risk in MFS (Salik and Rawla, 2016). Early diagnosis of the risk of aortic dissection, medical treatment to delay or prevent the progression of aortic dilation, and timely elective surgery have been shown to improve the prognosis in these patients (Gott et al., 1999; Erbel et al., 2014). Therefore, the primary objective for MFS healthcare professionals is to formulate a diagnosis that identifies patients suffering from aortic root dilation and determines possible surgical timing as early as possible (Erbel et al., 2014). In this regard, patients should perform echocardiography at the time of diagnosis and, 6 months later, determine the diameter of the aortic root and ascending aorta (Hiratzka et al., 2010). Individuals under 20 years of age with suggestive systemic MFS results but without cardiovascular involvement must undergo annual echocardiograms due to the potential risk of development of aortic disease (Erbel et al., 2014).

Several studies in recent years have highlighted the significant emotional and social impact of MFS on patients of all ages (Schneider et al., 1990; Peters et al., 2001; Johansen et al., 2013). Since MFS can manifest itself with a range of different phenotypic characteristics, the subjective experience of the disease is unique to each individual and requires educational and communicative models that adequately encompass this experience. It is crucial to create a forum to promote and critically discuss the possible educational and supportive models and experiences of dealing with the psychological and multifaceted challenges of people with MFS to address the current lack of evidence regarding the effectiveness of specific multi-strategy approaches on clinical outcomes of patients with MFS.

This discursive paper aims to synthesize and discuss the main psychosocial aspects and psychological needs in MFS and share the practical example of a counseling model, supportive services, and research activities of the psychological service available in the Cardiovascular-Genetic Center belonging to a reference center in northern Italy (i.e., IRCCS Policlinico San Donato). The paper highlights the importance of elucidating and sharing real-world educational, psychological, and psychotherapeutic support in practice to create awareness and analytical discussion among the various healthcare providers involved in the care program for people with MFS.

## 2. A practical example from Italy: the psychological service of the Cardiovascular-Genetic Center in a reference center for MFS

The Cardiovascular-Genetic Center of IRCCS Policlinico San Donato is a reference center for MFS in northern Italy. It involves multidisciplinary services by including cardiologists, clinical geneticists, nurse practitioners, and psychologists to offer comprehensive care for MFS patients during yearly (or planned) follow-ups at the outpatient clinic. Within the team, the psychologist is the professional with a pivotal role in assessing and planning psychological counseling, psychological advice to patients with MFS seeking pregnancy, psychological support or psychotherapy, support to physicians in enhancing their communication style (especially when communicating diagnosis), parental counseling, peer to peer groups activities, and research activities.

Psychological interventions focused on supporting patients with MFS have been included in a formal clinical pathway approved by the health management of the hospital. Precisely, the clinical pathway is specific to and targeted at patients diagnosed with MFS who carry out regular follow-up visits or who enter the Center for the first time, patients awaiting diagnosis who make the first visit or who are already followed by the Center, patients with other connective tissue disease or genetic arthropathies, children and adolescent patients, adults with MFS, and parents.

### 2.1. Psychological counseling, support, and psychotherapy

MFS patients often report having a significantly impaired body image, predicted by depression and anxiety (Hansen et al., 2020). When comparing operated and non-operated subgroups of MSF patients, it emerged that the operated group reported more alcohol consumption while the non-operated group had more sleep disturbances. When compared as a whole to the general population, the MFS patients appeared to have a moderate pain-related disability and mild depressive symptoms and sleep disturbances; however, they were more satisfied with their lives and considered themselves happier (Pólos et al., 2020).

Psychological counseling promotes treatment compliance and identifies the presence of psychological discomfort related to MFS. Precisely, a standardized assessment is planned through the clinical psychological interview and the use of targeted questionnaires, such as the General Anxiety Disorder 7-item scale (GAD-7) for the measurement of anxiety and the Patient Health Questionnaire-9 (PHQ-9) for the screening of depression (Giarelli et al., 2008; Löwe et al., 2008). When psychological signs and symptoms of distress are detected, the patients are directed toward adequate psychological or psychiatric pathways outside the Cardiovascular-Genetic Center. In any case, psychological counseling represents a moment of reflection on their illness history and processing emotions in a professional setting. If a patient reports suicidal thoughts or the possibility of self-harm, the psychologist notifies the Clinical Psychology Service of the hospital. During the sessions with pediatric patients, some issues with their caregivers could emerge, usually due to fears, worries, and doubts related to the disease. In these cases, the organization of psychological counseling sessions, including the parent or caregiver, is proposed.

The psychological sessions aim to explore and stimulate an adequate awareness of the patient suffering from MFS about the risks (if any) related to pregnancy, the probability of transmitting MFS to the fetus, and deepening the motivations that drive the couple to choose to pursue a pregnancy. Couples who decide to undertake a prenatal diagnosis course and pregnant mothers are offered the possibility of participating in psychological sessions, and the frequency is decided according to each case (approximately once a month). When psychological distress is reported by the patients (especially those who are territorially close to the center) they are offered psychological support or individual psychotherapy, depending on the specific needs and resources of the individual patient. These sessions allow for a more in-depth anamnesis to get to know the patients' life history beyond MSF and work on different topics. The sessions usually took place every fortnight face-to-face until the COVID-19 Health Emergency; from 2020, it was preferred to proceed through remote video calls, and as the pandemic emergency subsided, face-to-face sessions were resumed.

Among the most recurrent themes that emerged are: the management of anxiety, the acceptance of limits and diversity, the management of fears related to the unpredictability of the disease or the complications connected to it, and the re-elaboration of emotional experiences to illness and mourning. Once the objectives established with the patient have been achieved, the follow-up sessions will be carried out every 3 months and, subsequently, when required during the scheduled medical visits.

Specific tests and/or techniques are often used in psychological support sessions. Among the most used tests with patients with MFS or similar diseases, also reported in the literature, it was decided to administer mainly GAD7 and PHQ-9 because they are short, valid, and reliable. In addition, the SF-12 is also administered mainly for research purposes. Patients who report anxiety states related to the cardiovascular consequences of MFS (and which are also detected by the “GAD7” questionnaire) are offered cognitive and behavioral psycho-educational interventions, in which specific techniques of problem management are suggested and taught, together with breathing and relaxation techniques (for example progressive relaxation techniques and slow breathing).

All the described activities regarding psychological counseling, support and psychotherapy are well-rooted in the literature. In fact, research on patients with MFS has so far shown a reduction in the level of existential satisfaction (Velvin et al., 2016) with a significant impact on quality of life (Rao et al., 2016; Udugampolage et al., 2021) and on social relationships, associated with a negative perception of one's health level in both the physical and psychological dimensions (Fusar-Poli et al., 2008; Rand-Hendriksen et al., 2010). Patients with MFS appear to have significant anxiety levels (Benke et al., 2017) and depressive traits (Speed et al., 2017). Other studies have indicated a possible relationship between fatigue and chronic pain in patients with MFS (Bathen et al., 2014). Physical pain would, in fact, appear to be often present in patients with MFS (Bathen et al., 2014; Speed et al., 2017). Patients with MFS reported significantly higher fatigue scores and prevalence of severe fatigue compared to the general population and patients with rheumatoid arthritis, but lower than for other chronic conditions (Bathen et al., 2014). Executive function difficulties, particularly mental fatigue associated with MFS symptoms, were reported to affect QoL satisfaction and total QoL (Nelson et al., 2015; Ratiu et al., 2018).

There are also indications that awareness of aortic pathology can lead to emotional distress and a low assessment of life satisfaction (Staniši et al., 2018). In addition, patients undergoing life-saving cardiosurgical intervention experience higher levels of trait anxiety than healthy patients (Ghanta et al., 2016). For this reason, Benke and colleagues stress the importance of offering psychological or psychiatric interventions as required (Benke et al., 2017).

MFS also seems to influence patients' coping strategies, i.e., mechanisms that can regulate disturbing emotions and generate solutions to manage and solve the causes of stress. Difficulties in using coping mechanisms have also been found in patients with genetic diseases involving alterations in the aorta (Connors et al., 2015). It has also been pointed out that the subjective perception of discomfort does not always relate to the severity of the clinical-pathological expressions of the disease (Velvin et al., 2015), emphasizing the importance of psychological characteristics and coping mechanisms in the ability to accept and manage the disease.

When compared to a population with other chronic conditions (e.g., congenital heart disease), MFS patients reported an overall lower QOL, scoring significantly lower in the dimensions of pain/discomfort, anxiety/depression, mobility, and usual activities (Andonian et al., 2021). Nielsen and colleagues (Nielsen et al., 2019) also point out that MFS negatively impacts the patient's quality of life and that of family members, who, in turn, deserve support. A study indicated that QOL was affected significantly by social support, disease-related factors, and bio-behavioral factors (anxiety, depression, fatigue, pain, and body image), with the bio-behavioral factors having the strongest and most direct effect on QOL (Moon et al., 2016). In another study that investigated a large cohort of patients with MFS (389 adults), HRQOL was below the population norm. Better QOL was independently associated with socioeconomic factors, mainly insurance status, employment, and not factors related to general health or MFS severity (Goldfinger et al., 2017). Another study confirmed this trend, in which Hereditary Thoracic Aortic Diseases patients (mainly MSF) reported subnormal HRQOL, especially in females. Also, in this case, for both males and females, factors such as employment, coping style, and disease acceptance were confirmed to be more important for HRQOL than disease-related factors (Thijssen et al., 2020).

When it comes to pediatric patients, it is also essential to provide psychological support to the parents, whether or not they, too, are MFS patients. In fact, feelings of guilt and difficulties in accepting and managing the disease are frequent issues for the parents of our young patients. Furthermore, parents must be helped to understand how they can support their child in daily difficulties, recognizing the limits of the disease in the social and school environment. The parental counseling sessions aim to raise awareness among MFS parents about the importance of pediatric patients understanding what is happening around them in relation to their health. The psychologist supports the parents by giving suggestions on how to provide information and explanations to their children (regarding diagnosis, treatments, evolution, and consequences of the disease on future life). Depending on the case, the parents are provided with a fairy tale (for children under 6 years) or a comic book (for children between 7 and 12 years old) created by the psychologist of the Center to support them in the explanation of MFS and provide ideas to facilitate communication on the theme of the disease. Sometimes, if the parent has doubts about how and when to talk to their child about MFS, the possibility of supporting the parents to communicate with the child at the end of medical examinations can also be proposed.

Another relevant activity in the experience of the psychological service of the Cardiovascular-Genetic Center in a reference center for MFS is the facilitated peer-to-peer support groups. Self-help groups, aimed at patients and family members, are organized monthly and are organized by the Center's psychologist every month. In the meetings, no specific topics are addressed, but there is a focus on the themes that emerge spontaneously based on the stories and experiences of the individual participants. In fact, the objective is to share one's own experience and to discuss possible solutions to the difficulties they are experiencing. The psychologist plays the role of facilitator, enabling the possibility of expression of all participants and valuing and emphasizing positive strategies. The facilitated peer-to-peer support groups have been active since 2015: the groups had always taken place face-to-face until 2020 when, due to the COVID-19 Health Emergency, it was decided to continue online via videoconference. This has made it possible to expand participation to patients located throughout the Italian territory. To date, 70 monthly groups have been organized, with an average of about eight participants per group, with a fairly balanced percentage of men and women. On the other hand, online groups have an average of about 15/16 people per meeting, of which at least 80% are women.

Considering the perspective of clinicians, another initiative is dedicated to the support of the physicians at the time of communication of the diagnosis. This activity occurs when the physician (cardiologist and/or geneticist) considers that the patient needs psychological support at the delicate moment of diagnosis communication. This need can also be seen moments after the diagnosis, for example, when the hypothesis of cardio-surgical intervention is proposed to a patient known to our Center.

### 2.2. Research activities

Alongside the clinical activity, the psychologists are also promoters of specific research activities on the psychological aspects of the patients of the Vascular CardioGenetic Center. Precisely, research has been focusing on the quality of life and coping mechanisms of adult patients and the wellbeing of pediatric patients. Thus far, two types of research have been approved by the ethics committee of reference with the following objectives: one research aims to understand the impact that the clinical phenotypic manifestations of MSF have on psychosocial aspects, self-esteem, subjective perception of the disease and coping mechanisms in adult patients and to identify the factors involved in motivating the initiation of psychotherapy or psychological support in those affected by MSF. The other research focuses on assessing Italian children and adolescents with MFS (3–17 years) quality of life. All subjects participating in both studies are recruited during the follow-up visits at the Vascular CardioGenetic Center of the hospital. For data collection, the psychologist is responsible for carrying out small structured interviews and administering self-report questionnaires aimed at understanding the impact of MFS on mental health and quality of life. These results activities are consistent with the current trends emerging from the literature.

In the literature, there is a general agreement on the importance of adopting a multidisciplinary approach to patient care with MFS, in which the psychological effects of the disease are considered (Kondylakis et al., 2013; Caruso et al., 2018). In general, a personalized approach in healthcare is possible when there are efforts to create a patient profile that takes into consideration as many psychosocial elements as possible (Kondylakis et al., 2013) and when there is also consideration of the possible role of emotions in decision-making processes regarding the handling of health conditions (Mazzocco et al., 2019).

Moon and colleagues (Moon et al., 2016) suggest providing intervention programs that improve social support as a strategy to counter bio-behavioral changes such as depression and improve the quality of life of patients with MFS. In addition, studies not only referring to MFS underline the importance that health professionals consider medical treatment along with psychosocial aspects that accompany patients throughout their lives, suggesting the treatment of important topics in daily disease management (Giarelli et al., 2008; Treasure et al., 2018).

In particular, the importance of helping the patient and the family to deal with the diagnosis of MFS and its various manifestations is underlined (Giarelli et al., 2008). According to Giarelli and colleagues (Giarelli et al., 2008), it is also essential to help young patients, through parents' support, become gradually responsible for their own treatments and accept the limits related to physical activity since this will positively affect their adaptation to the disease. The importance of openly and comprehensively communicating the disease's evolution to young patients and their caregivers were also stressed by addressing its concerns, as this could help reduce stressors (Neville, 1998; Casiday, 2006).

With reference to MFS, Treasure and colleagues recommend that healthcare professionals support patients in their decision-making and choice processes, such as prophylactic aortic root surgery (Treasure et al., 2018). Another important topic that warrants attention is the possible transmissibility of MFS by pregnant women; for this reason, it is suggested to discuss issues about pregnancy planning and the possible risks so that there is a sufficient degree of awareness among the couples who could be affected by fears, doubts, and possible genetic transmission to the offspring (von Kodolitsch et al., 2015). In a recent review of psychosocial aspects related to MFS, Nielsen and colleagues highlight the importance of appropriate interventions for young MFS patients of all age groups (Nielsen et al., 2019). During childhood, the disease can interfere negatively with social interactions. In qualitative research investigating the life experiences of MSF patients through interviews, the following themes were identified; “difficulties in keeping up with peers” and “being and feeling different from peers” (Warnink-Kavelaars et al., 2019).

In adulthood, other problems can have a negative impact on the quality of life, such as physical pain, which in turn also affects work participation. Psychological and psychiatric distress can also develop due to the complexity of the disease (Fusar-Poli et al., 2008). Psychological or psychotherapeutic support is essential given the variability of the clinical expression and symptoms of MFS and the impact of the disease on many aspects of the person's life. A critical literature review of the psychiatric and neuropsychological issues in MFS concluded that these patients should carefully assess their psychological and neuropsychological domains, suggesting that some might require specific rehabilitation programs. The authors stress the importance of adopting a multidisciplinary approach (Gritti et al., 2015).

Although some studies stress the importance of offering psychological support to patients who have undergone cardio-surgical interventions (Benke et al., 2017; Benninghoven et al., 2017) and Nielsen and colleagues recently highlighted the need to provide support focused on coping strategies for both the patient and family members (Nielsen et al., 2019), to date, the literature lacks specific indications, guidelines or structured experiences that also consider the importance of psychosocial variables for the clinical treatment of these patients (Velvin et al., 2015). For Nielsen and colleagues (Nielsen et al., 2019), understanding the psychosocial implications of MFS is key to designing therapies that address stress and develop patients' psychological and emotional wellbeing, thus improving their quality of life. It is also pivotal to help patients manage pain, improve their physical and mental health, and create information and support groups for family members to avoid the risk of burnout. In this context, sharing structured experience and practical examples of organizing psychological services for patients with MFS might facilitate increased awareness of which elements might require more attention from the international scientific arena and facilitate positive cross-contamination of good practice.

### 2.3. Limitations

In this work, we aimed to comprehensively describe a practical example of psychological service for patients with MFS. However, we recognize certain limitations that warrant disclosure. Firstly, this manuscript does not evaluate the service's effectiveness quantitatively, limiting the generalizability and applicability to other contexts. Future research should assess the service's efficacy in improving the mental health and wellbeing of patients with MFS using appropriate research designs, such as randomized controlled trials. Secondly, our work does not thoroughly compare the proposed service with existing services or interventions. A more in-depth literature review and comparison with alternative approaches could offer valuable insights into potential advantages. In fact, integrating our findings with the broader literature on psychological interventions for MFS would help contextualize our work within the existing body of knowledge. Additionally, we acknowledge the need for further research on long-term outcomes in real-life practice. Evaluating the long-term impact of psychological services on patients' wellbeing, mental health, and overall functioning would provide a more comprehensive understanding of their effectiveness.

Despite the successful implementation of psychological support services at the Cardiovascular-Genetic Center of IRCCS Policlinico San Donato, challenges remain in providing comprehensive psychological care for MFS patients. The primary challenge lies in addressing their diverse psychological needs, influenced by factors like age, disease severity, socioeconomic status, and individual characteristics. The effectiveness of psychological support may also vary based on patients' adherence to recommended therapies, engagement in support groups, and resource availability. Therefore, more real-world data is necessary. Notably, the limited research, especially in the pediatric population, underscores the need for further investigation into MFS's psychological aspects. In the current landscape, it is vital to emphasize the importance of collaboration among healthcare professionals in a multidisciplinary team to ensure that MFS patients' psychological needs are adequately addressed and integrated into their overall care plan.

## 3. Conclusions

Although psychological interventions should be integrated into the care for patients with MFS, there is still a lack of available structured experiences of dedicated services to meet the psychological needs of these patients, as well as available evidence in this regard. This discursive paper provided an overview of the current knowledge of psychosocial characteristics and support strategies for patients with MFS and aimed to share an example of how psychological services might be embodied in general care.

The psychological services offered to MFS patients are meant to support the patient and family members, reducing the psychological distress associated with the disease while promoting an adaptation to the disease that guarantees a good quality of life. The psychology service represents a space where patients can find acceptance and be heard in regard to emotions that are often not shared with medical staff. Psychological services are, in fact, a tool to support the medical team that is faced with the complexity of a patient in which emotional aspects play a fundamental role in adaptation to the disease and compliance with treatments.

One advantage of a psychology service that supports the medical follow-up path is to ensure continuity in the support and psychological help for these patients. The psychology service of the analyzed example represents a reference point for listening to and treating psychological needs related to MFS, needs that for the patient sometimes assume the same importance as those related to medical treatment. In particular, regarding patients of developing age, this support aims to promote greater knowledge and adaptation to the disease, favoring compliance with treatments through the help of parents. For patients in childhood and pre-adolescence, it is useful to create tools such as fairy tales or comics that can help them understand MFS and what it entails. For adolescent patients, developing information material that explores the importance of respecting limits in physical activities and representing possible risks and complications could be helpful.

An internal psychology service at the Center that specializes in treating patients with MFS also promotes meetings and communication between patients, ensuring that patients can avoid social isolation, which these patients often experience. In this sense, self-help groups represent an opportunity for patients, family members, and caregivers to socialize and feel less isolated.

## Author contributions

Conceptualization and writing—original draft preparation: MP, AB, NU, and EC. Methodology: RC, AP, and JT. Resources: AP and NU. All authors have read and agreed to the published version of the manuscript.
